# ONC206, an Imipridone Derivative, Induces Cell Death Through Activation of the Integrated Stress Response in Serous Endometrial Cancer *In Vitro*

**DOI:** 10.3389/fonc.2020.577141

**Published:** 2020-10-20

**Authors:** Yingao Zhang, Yu Huang, Yajie Yin, Yali Fan, Wenchuan Sun, Xiaoling Zhao, Katherine Tucker, Allison Staley, Sarah Paraghamian, Gabrielle Hawkins, Varun Prabhu, Joshua E. Allen, Chunxiao Zhou, Victoria Bae-Jump

**Affiliations:** ^1^Division of Gynecologic Oncology, University of North Carolina at Chapel Hill, Chapel Hill, NC, United States; ^2^University of North Carolina School of Medicine, Chapel Hill, NC, United States; ^3^Department of Gynecologic Oncology, Chongqing University Cancer Hospital, Chongqing, China; ^4^Department of Obstetrics, Beijing Obstetrics and Gynecology Hospital, Capital Medical University, Beijing, China; ^5^Oncoceutics, Philadelphia, PA, United States; ^6^Lineberger Comprehensive Cancer Center, University of North Carolina at Chapel Hill, Chapel Hill, NC, United States

**Keywords:** ONC206, serous endometrial cancer, cellular stress, proliferation, invasion

## Abstract

ONC206 (Oncoceutics) is an imipiridone with nanomolar potency and analogue of ONC201, a selective dopamine receptor D2 (DRD2) antagonist currently being investigated in phase II clinical trials for serous endometrial cancer (SEC). This study investigated the anti-proliferative efficacy of ONC206 in SEC cell lines as well as its impact on cellular stress and adhesion/invasion. ONC206 inhibited cellular proliferation in a dose-dependent manner and was more potent than ONC201 in the ARK1 (IC_50_ = 0.33µM vs. IC_50_ = 1.59uM) and SPEC-2 (IC_50_ = 0.24uM vs. IC_50_ = 0.81uM) cell lines. Treatment with ONC206 resulted in induction of ROS production and reduction of mitochondrial membrane potential, accompanied by an increase in cleaved caspase-3 and caspase-9 activity (p < 0.01). ONC206 also significantly inhibited cellular adhesion and migration in both cell lines (p < 0.01). Pretreatment with the stress inhibitor N-acetylcysteine (NAC) significantly attenuated the efficacy of ONC206 on cell proliferation, ROS production and cellular invasion. ONC206 demonstrates nanomolar potency for the inhibition of proliferation in SEC cells. Specifically, ONC206 utilizes ISR activation as a significant pathway in the propagation of its anti-proliferative and anti-metastatic effects. Thus, ONC206 may be a promising agent in future SEC clinical trials as was its predecessor ONC201.

## Introduction

With over 65,000 new cases estimated in 2020, endometrial cancer (EC) remains the most common gynecologic malignancy in the United States (1). Within these cancers, 80%–90% have an endometrioid adenocarcinoma histology and are classified as type I ECs. Type II ECs encompass all other histological subtypes (i.e., serous, clear cell, and carcinosarcoma); although less prevalent, these tumors behave much more aggressively in terms of disease progression and recurrence rates after treatment (2). Serous EC (SEC) is a type II EC that comprises approximately 10% of all cases but contributes to up to 40% of deaths in all EC patients (3). At the time of diagnosis, up to 70% of SEC patients exhibit extrauterine disease spread compared to less than 5% of patients with endometrioid ECs, which likely contributes to the poorer observed outcomes (3–6). Reported 5-year survival rates for SECs are dismal, ranging from 30% to 55% (3, 5, 7), compared to 75% for women with high grade endometrioid tumors and >90% for low grade endometrioid tumors (7–9). This highlights a continuing need to explore novel therapeutic strategies for SEC, an especially deadly histology of EC (10).

ONC206 (Oncoceutics) is an imipiridone small molecule with nanomolar potency and an analogue of ONC201, a selective bitopic dopamine receptor D2 (DRD2) antagonist currently being investigated in phase II clinical trials for SEC. ONC201 was originally identified in a screen for molecules that increased the activity of tumor necrosis factor (TNF)–related apoptosis-inducing ligand (TRAIL), a cytokine heavily involved in tumor-specific cytotoxicity with minimal involvement of nonneoplastic cells (11, 12). Recent studies have shown that ONC201 exhibits anti-tumorigenic effects in pre-clinical models of solid tumors and hematologic malignancies through upregulation of TRAIL and activation of the integrated stress response (ISR) combined with inactivation of AKT/ERK and other pro-survival signaling pathways in a p53-independent manner (13–16). Several phase I and II clinical trials found that ONC201 is well tolerated and has initial clinical proof of activity in advanced stage cancer patients (17–20). ONC206 has also shown anti-tumor effects *in vitro* across multiple cancers types, most notably glioblastoma, melanoma, and colorectal cancer, as well as EC (21, 22). ONC206 is more potent to suppress oxidative phosphorylation, regulate apoptosis mediators, and inhibit the activity of AKT/ERK pathway as compared to ONC201 in cancer cells. Through similar mechanisms to ONC201, ONC206 shows an improved inhibition of cell proliferation and invasion *in vitro* (21, 22). As our group had previously shown that ONC201 inhibited proliferation in SEC cell lines (13), the aim of this study was to expand on this work and evaluate the effect of ONC206 on cell proliferation, cellular stress, migration, and invasion in SEC cells. Our results demonstrate that ONC206 exhibited more potent anti-proliferative activity than ONC201 *in vitro*. Moreover, we found that activation of the ISR remains a mechanistic keystone in the overall anti-neoplastic and anti-metastatic potentials of ONC206 in SEC cells.

## Methods and Materials

### Cell Culture and Reagents

Two SEC cell lines, SPEC2 and ARK1, were used for all experiments. SPEC2 cells were maintained in DMEM/F12 medium with 10% fetal bovine serum (FBS). ARK1 cells were maintained in RPMI 1640 medium with 10% FBS. All media was supplemented with 100 U/ml of penicillin and 100 ug/ml of streptomycin. The cells were cultured in humidified 5% CO_2_ environment at 37°C. ONC201 and ONC206 were obtained from Oncoceutics, Inc. MTT [(3-5-dimethylthiazol-2-yl)-2,5-diphenyltetrazolium bromide] was purchased from Sigma (St. Louis, MO), and maintained in dimethylsulfoxide (DMSO). All antibodies were purchased from Cell Signaling Technology (Beverly, MA). Enhanced chemiluminescence western blotting detection reagents were purchased from Amersham (Arlington Heights, IL). All other chemicals were purchased from Sigma.

### MTT Assay

SPEC2 and ARK1 cells were plated and grown in 96-well plates at a concentration of 3–5 × 10^3^ cells/well for 24 h. The cells were then treated with varying doses of either ONC201 or ONC206 for 72 h. MTT (5 mg/ml) was added at 5 ul/well at the end of treatment. After 1 h of incubation, the MTT reaction was terminated through the addition of DMSO at 100 ul/well. The results were read by measuring absorption at 575 nm with a microplate reader (Tecan; Morrisville, NC). The effects of ONC201 and ONC206 were calculated as a percentage of control cell growth obtained from DMSO-treated cells grown in the same 96-well plates. Each experiment was performed in triplicate to assess for consistency of results.

### Cleaved Caspase-3 and Caspase-9 ELISA Assays

Caspase activity assays were performed with some modifications as previously described (23). In brief, the SPEC2 and ARK1 cells were plated in 6-well plates at a concentration of 2.5 × 10^5^ cells/well for 24 h. The cells were treated with ONC206 at different concentrations for 24 h. 150–180 ul 1X caspase lysis buffer was added to each well. Protein concentration was determined using the BSA assay. Lysates (10–15 ug) in a black clear bottom 96-wells were incubated with reaction buffer and 200 uM of caspase substrates for 30 min. The fluorescence of each well was determined using a microplate reader (Tecan, Morrisville, NC). The selective substrates Ac-DEVD-AMC and Z-IETD-AFC (AAT Bioquest, Sunnyvale, CA) were used for caspase-3 and caspase-9, respectively. Each experiment was repeated three times to assess for consistency of results.

### Reactive Oxygen Species Assay

The SPEC2 and ARK1 cells were plated and grown in 96-well plates at a concentration of 8-12 × 10^3^ cells/well for 24 h. The cells were then treated with varying doses of ONC206 for an additional 24 h. To measure intracellular ROS production, cells were exposed to the oxidation sensitive probe 2’,7’-dichlorofluorescin diacetate (DCFH-DA) at 10 uM for 1 h. Fluorescence was then determined at 575 nm using a microplate reader (Tecan) and normalized to corresponding MTT measurements of the same plate.

### Mitochondrial Membrane Potential Assay

Mitochondrial membrane potential was analyzed using the specific fluorescent probes JC-1 (AAT Bioquest, Sunnyvale, CA). The SPEC2 and ARK1 cells were plated and treated with different concentrations of ONC206 for 8 h. Treated cells were then incubated with 2 uM JC-1 for 30 min at 37°C. The levels of the fluorescent probes were measured using a Tecan plate reader at two excitation/emission wavelength pairs. Each experiment was repeated three times to assess for consistency of results.

### Adhesion Assay

Each well in a 96-well plate was coated with 100 ul of laminin-1 (10 ug/ml) and allowed to incubate at 37°C for 1 h. The fluid was then aspirated and 200 ul/well of blocking buffer was added for 45 min at 37°C. Wells were then washed with PBS and placed on ice. After resuspension in PBS and varying concentrations of ONC206, SPEC2, and ARK1 cells were added to the wells at 2500 cells/well and allowed to incubate for 1 h at 37°C. The media were then aspirated and the cells were fixed by addition of 5% glutaraldehyde at 100 ul/well for 30 min at room temperature. Adhered cells were then washed with PBS and stained with 0.1% crystal violet solution at 100 ul/well for 30 min. Each well was washed with sterile water, and 100 ul/well of 10% acetic acid was added to solubilize the dye. Absorbance was measured at 575 nm using a microplate reader (Tecan) after 5 min of plate shaking. Experiments were performed in triplicate to assess for consistency.

### Transwell Assay

Cell invasion assays were performed using a 96-well plate coated with 0.5–1 × BME. The ARK1 and SPEC2 cells were starved for 12 h and then seeded in the upper chambers of the wells and the lower chambers were filled with regular medium and differing concentrations of ONC206 (0.05, 0.5, and 5 uM). The plates were incubated for 3.5 h to allow invasion into the lower chambers. After washing the upper and lower chambers with PBS, 100 ul of calcein AM solution (Invitrogen, Carlsbad, CA) was added to the lower chambers and incubated for 30–60 min. The lower chamber plate was measured by plate reader for reading fluorescence at EX/EM 485/520 nm. Each experiment was repeated twice.

### Wound Healing Assay

SPEC2 and ARK1 cells were plated in 6-well plates at a concentration of 3–5 × 10^5^ cells/well and grown for 24 h or until >80% confluency assessed by microscopy. Uniform wounds were created in each plate through the cell monolayer using a 20-ul pipette tip. Cells were then washed and treated with different concentrations of ONC206 for 24 h. Pictures were taken at 24, 36, 48, and 72 h post-treatment. Photos and wound sizes were measured and analyzed using the software ImageJ (National Institutes of Health (NIH); Bethesda, MD). Experiments were performed in triplicate to assess for consistency.

### Western Immunoblotting

SPEC2 and ARK1 cells were plated in 6-well plates at a concentration of 2–3 × 10^5^ cells/well and grown for 24 h or until 60%–70% confluency assessed by microscopy. Cells were then treated with ONC206 for 20–30 h. Cell lysates were prepared in radioimmunoprecipitation assay (RIPA) buffer and isolated protein solutions were maintained on ice. Equal amounts of protein were separated by gel electrophoresis and transferred onto a polyvinylidene difluoride (PVDF) membrane. Membranes were blocked with a 5% nonfat milk solution and then incubated with a 1:1,000 dilution of primary antibodies overnight at 4°C. Membranes were then washed and incubated with a secondary peroxidase-conjugated antibody for 1 h. Antibody binding was detected using an enhanced chemiluminescence detection system on the Alpha Innotech Imaging System (Protein Simple; Santa Clara, CA). After developing, the membranes were stripped and re-probed using anti-ß-actin or anti-α-tubulin antibodies to confirm equal protein loading. Intensity for each band was measured and normalized to either B-actin or a-tubulin as an internal control. Experiments were performed in duplicate to assess for consistency.

### Stress Inhibition With N-Acetylcysteine

MTT, ROS, and wound healing assays, along with western immunoblotting, were repeated with additional NAC experimental groups. For each assay, cells were pretreated with 3 uM of NAC for 3 h and then washed with PBS prior to drug treatment with difference concentrations of ONC206. All other variables including cell plating density, drug concentration, and other assay-specific protocols were held constant as detailed above. For consistency, MTT, ROS, and wound healing assays were performed in triplicate, and western immunoblotting was performed in duplicate.

### Statistical Analysis

Data are given as the mean ± SD. Statistical significance was analyzed by the two-sided unpaired Student’s t-test from at least three replicates. GraphPad Prism 6 (La Jolla, CA USA) was used for all graphs and significance tests. P values of <0.05 were considered to have significant group differences.

## Results

### ONC206 Inhibits Cellular Proliferation in SEC Cells

**Figure 1:**
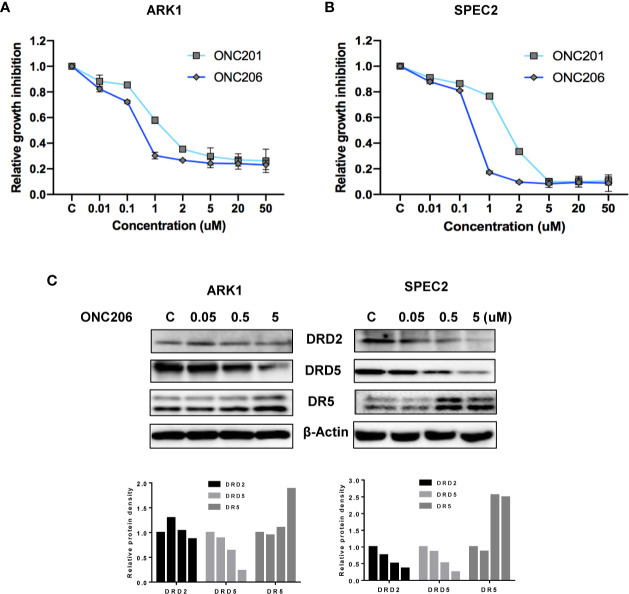
ONC206 inhibited cellular proliferation in SEC cells. Proliferation was measured by MTT assays, and the effect of ONC206 on the cellular expression of dopamine receptor D2 (DRD2), dopamine receptor D5 (DRD5), and death receptor 5 (DR5) was measured by Western immunoblotting. **(A)** In the ARK1 cells, ONC206 exhibited a dose-dependent effect on cellular proliferation and showed increased potency when compared to ONC201 (IC50 = 0.33uM vs. IC50 = 1.59uM). **(B)** The same effect was seen in the SPEC-2 cells (IC50 = 0.24uM vs. IC50 = 1.81uM). **(C)** Representative Western immunoblots demonstrated the dose-dependent effect of ONC206 on decreasing both DRD2 and DRD5 expression while increasing DR5 levels in both cell lines. β-actin served as an intern control. All experiments were performed in triplicate.

The SEC cell lines, ARK1 and SPEC-2, were cultured in medium with various concentrations of ONC201 or ONC206 for 72 h and cellular viability was analyzed with the MTT assay. As shown in **Figures 1A, B**, ONC206 exhibits dose-dependent effects on cellular growth for both cell lines at significantly lower doses than that of ONC201. The mean IC50 values of ONC201 were 1.59 uM for ARK1 and 1.81 uM for SPEC-2; the mean IC50 values of ONC206 were 330 nM for ARK1 and 240 nM for SPEC-2.

Because ONC201 induces cytotoxicity through the DRD2/5 and TRAIL/DR5 pathways, we examined expression of key targets of these pathways after treatment with ONC206. As illustrated in **Figure 1C**, western immunoblotting showed that ONC206 significantly increased levels of DR5 protein expression and decreased the expression of DRD2 and DRD5 in both cell lines after 24 h of treatment with ONC206. These results suggest that ONC206 inhibits cell proliferation through DRD2/5 and TRAIL/DR5 pathways and shows more cytostatic function compared with ONC201 in SEC cells.

### ONC206 Induces Apoptosis in SEC Cells

**Figure 2:**
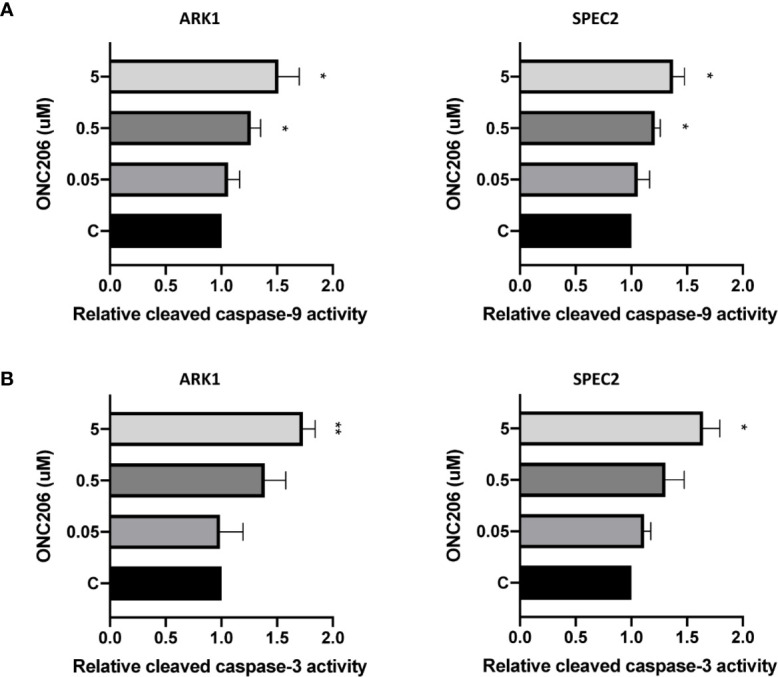
ONC206 induced apoptosis in SEC cells. Cleaved caspase-3 and caspase-9 activity was measured as a direct indicator of apoptotic activity using ELISA assays. **(A)** Both cell lines showed a significant dose-dependent increase in relative cleaved caspase-9 activity to ONC206 treatment (p = 0.02–0.04). **(B)** The same effect was seen with caspase-3, though only significant at higher doses of ONC206 (p = 0.008–0.02). All experiments were performed in triplicate. *p < 0.05, **p < 0.01.

In order to characterize the utilization of apoptotic pathways by ONC206, we performed ELISA assays for cleaved caspase-3 and caspase-9 proteins. We treated ARK1 and SPEC-2 cells with varying concentrations of ONC206 for 24 h and found a dose-dependent increase in the cleaved activities of caspase-3 and caspase-9 when compared to controls (**Figures 2A, B**). Treatment with ONC206 (0.05–5 uM) significantly induced cleaved caspase-3 and caspase-9 activities; at a dose of 5 uM, ONC206 increased activity levels by 1.7- and 1.5-fold respectfully in ARK1 cells (p = 0.008–0.04), and by 1.7- and 1.4-fold respectfully in SPEC-2 cells (p = 0.02–0.03). These results indicate the involvement of DR5-induced caspase-dependent pathways in the inhibition of proliferation in SEC cells treated with ONC206.

### ONC206 Activates the Integrated Stress Response

**Figure 3:**
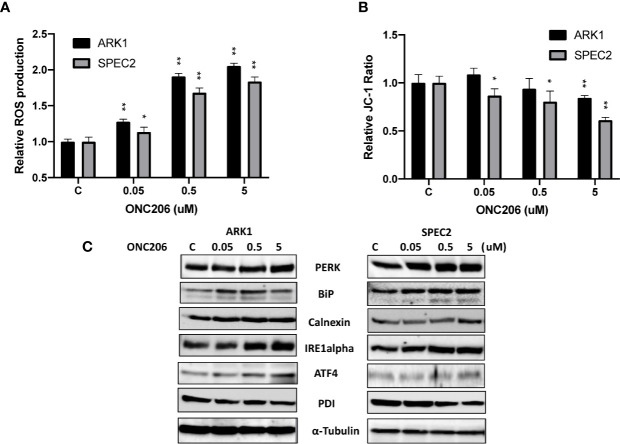
ONC206 induced the integrated stress response in SEC cells. JC-1 fluorescence was utilized as an inverse indicator of mitochondrial stress, while intracellular ROS levels directly correlated to general cellular stress levels. **(A)** ROS production was significantly increased at all doses of ONC206 in both cell lines (p < 0.001). **(B)** ONC206 significantly reduced mitochondrial membrane potential in both SEC cell lines. **(C)** Representative Western immunoblots demonstrated dose-dependent increases in the intracellular stress markers PERK, BiP, calnexin, IRE1-alpha, AIF-4, as well as decreases in the inverse marker PDI. All experiments were performed in triplicate. *p < 0.05, **p < 0.01.

Given that ONC201 activates the ISR in many types of solid cancers, we examined the effects of ONC206 on cellular stress and mitochondrial membrane potential using DCFH-DA and JC-1 assays, respectively. After a 24-h treatment with varying concentrations of ONC206, a dose-dependent response on reactive oxygen species (ROS) assays can be seen, illustrating the effects of ONC206 on inducing intracellular stress (**Figure 3A**). ROS was increased by 83%–105% from baseline in ARK1 and SPEC-2 cells at a dose of 5 uM after 24 h of treatment. **Figure 3B** shows significantly decreased mitochondrial membrane potential of 16%–38% in ARK1 and SPEC-2 cells after treatment with ONC206 for 18 h at 5 uM.

To further assess the role of cellular stress in ONC206-treated SEC cells, we used western blotting to detect changes of ISR-specific proteins. After treatment with ONC206 for 24 h, we found increased expression of PERK, BiP, calnexin, ATF4, IRE-alpha, and decreased PDI in both cell lines (**Figure 3C**). These results support our hypothesis that cellular stress may be a major mechanism for the inhibition of cellular proliferation by ONC206.

### ONC206 Inhibits Cellular Adhesion and Invasion *In Vitro*

**Figure 4:**
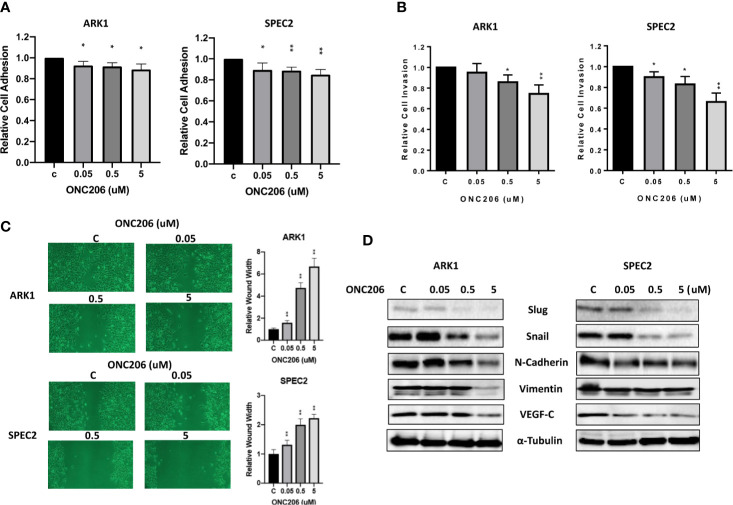
ONC206 inhibited cellular invasion and adhesion in SEC cells. **(A)** In both the ARK1 and SPEC-2 cell lines, cellular adhesion was significantly impaired by increasing concentrations of ONC206 (p = 0.01–0.02). **(B)** ONC206 decreased invasive ability in both cell lines in a dose dependent manner. **(C)** Invasion potential was drastically reduced at all tested concentrations of ONC206; at 5 uM, relative wound healing was decreased more than 2-fold in the SPEC-2 cells and over 7-fold in the ARK1 cells (p < 0.001). **(D)** Representative Western immunoblots demonstrated that ONC206 decreased expression of proteins involved in EMT, or epithelial-mesenchymal transition (slug, snail, E-cadherin, vimentin) as well as markers of angiogenesis (VEGF-C). All experiments were performed in triplicate. *p < 0.05, **p < 0.01.

To assess the effects of ONC206 on the adhesion and invasion potential of SEC cells, laminin adhesion, transwell invasion, and wound healing assays were used. After a 1-h treatment with varying concentrations of ONC206, both ARK1 and SPEC-2 cells showed significant inhibition of cellular adhesion at all drug concentrations (**Figure 4A**). Transwell assay results showed that the invasive capacity of the ARK1 and SPEC-2 cells was reduced by ONC206 treatment in a dose- dependent manner. ONC206 (5 uM) significantly reduced the invasive ability of the ARK1 and SPEc-2 cell lines by 26.6% and 34.0% (**Figure 4B**). Scratch wound healing assays measured the extent of cellular migration into the scratched or “wounded” areas after a 48-h treatment with ONC206. Both ARK1 and SPEC-2 cells showed a dose-dependent inhibition of migration back into the wounded areas. At a dose of 5 uM, ONC206 significantly increased wound width from baseline by more than 2 times in SPEC-2 cells (p < 0.001) and almost 7 times in ARK1 cells (p < 0.001) (**Figure 4C**).

Because various cellular membrane and cytoskeletal assembly proteins are involved in the adhesive and invasive potential of cancer cells, we further analyzed the effects on ONC206 on the epithelial-mesenchymal transition (EMT) and angiogenesis in SEC cells. After treating the cells with ONC206 at various concentrations for 24 h, western immunoblotting illustrates a downregulation of proteins Slug, Snail, E-Cadherin, Vimentin, as well as VEGF-C (**Figure 4D**). These results further support the role of ONC206 in its effects on decreasing invasion and angiogenic potential in SEC cells.

### ONC206 Inhibits Cell Proliferation and Invasion Dependent on Cellular Stress Pathway

**Figure 5:**
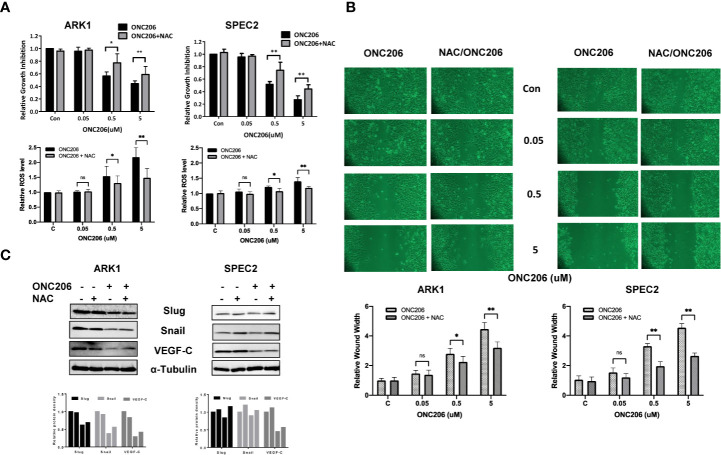
NAC reversed effects of ONC206 on proliferation and invasion in SEC cells. Prior to treatment with ONC206, cells were pre-treated with 3 uM of the stress-inhibitor NAC for 3 h. **(A)** Pre-treatment with NAC attenuated the effect of ONC206 on proliferation and ROS in both the ARK1 and SPEC-2 cell lines. **(B)** Additionally, wound healing potential induced by ONC206 was also significantly increased after pre-treatment with NAC. **(C)** Representative Western immunoblotts suggest that the combination of NAC and ONC206 significantly decreased the expression of Slug, Snail, and VEGF compared to ONC206 or NAC alone. All experiments were performed in triplicate. *p < 0.05, **p < 0.01, ns: no significance.

To verify ISR activation as one of the major mechanisms through which ONC206 exerts its downstream effects, we used NAC, a known cellular stress inhibitor, to pretreat the ARK1 and SPEC-2 cells before exposure to ONC206. Prior to the addition of ONC206, cells were pre-treated with 3 uM of NAC for 3 h. In both the ARK1 and SPEC-2 cells, exposure to NAC attenuated the effects of ONC206 on both cellular proliferation as well as intracellular ROS production, particularly in the 500 nM to 5 uM range (**Figure 5A**). NAC also reversed the effects of ONC206 on the inhibition of wound healing in both cell lines (**Figure 5B**). Lastly, western immunoblotting demonstrated that NAC exposure reduced ONC206-mediated downregulation of EMT proteins, Slug, and Snail (**Figure 5C**). These results help elucidate ISR activation as one of the main pathways through which ONC206 induces cell death in SEC cells.

## Discussion

SEC often presents as a clinical challenge due to its propensity for aggressive extrauterine spread and high recurrence rates, and unfortunately, there are limited options for effective second-line treatments (24, 25). ONC201 is a first-in-class small molecule selective DRD2 antagonist that is in Phase II clinical trials in select advanced cancers, including type 1 and 2 ECs, after completion of a promising first-in-man clinical trial in advanced solid tumors that included EC patients (20). ONC206 is a chemically modified derivative of ONC201 that was created to improve on the anti-tumorigenic effects of its predecessor (21, 22). In our current study and in line with our hypothesis, when compared to its parent compound ONC201, ONC206 shows similar anti-tumor activities including DR5 targeting, induction of apoptosis, and inhibition of cellular invasion. Additionally, ONC206 exhibited more potent anti-proliferative activity than ONC201 in SEC cells, implying that ONC206 may have stronger anti-tumor potential.

ONC206 operates through similar mechanisms of action of ONC201 but has increased potency, leading to increased antitumor activity, demonstrated through our comparisons with ONC201, and in line with current literature (13, 21, 22). For glioblastoma, ONC206 has been found to be more effective in reducing the expression of pro-apoptotic Bcl-2 family members in U87 and stem-like glioblastoma cells compared to ONC201 (21). In addition, ONC206 *versus* ONC201 was more efficacious in reducing ATP levels, suppressing glycolysis and oxidative phosphorylation, resulting in a transcriptional state of energy starvation and activation of ER-stress related pathways (21). Induction of apoptosis by ONC201 mainly depended on activation of ISR that transcriptionally regulate DR5 and TRAIL (26). The sensitivity of human tumor cells to ONC201 is associated with induction of DR5 in an ATF4 and CHOP-dependent manner, indicating that ER stress controls the process of apoptosis induced by ONC201 (14, 15).

Our findings demonstrated that ONC206 increased the expression of ER stress-related proteins including ATF4, PERK, and Bip, and significantly inhibited tumor cell migration and invasion *in vitro*. We then introduced NAC in order to block the cellular stress pathway and found that the inhibition of stress with NAC partially attenuated the effects of ONC206 on both cellular proliferation and invasion, in line with our hypothesis that cellular stress pathways are intimately involved in imipridone-mediated cell proliferation and invasion. Thus, our results clarified a major mechanistic step for ONC206 in its anti-tumorigenic effects potentially in SEC.

Over the last decade, there have been significant advances in the research of targeted therapies for gynecologic malignancies, perhaps most notably the development of PARP-inhibitors for ovarian cancer, amongst many others (27). However, thus far, there have only been two FDA-approved non-hormonal therapies (pembrolizumab in NCT02054806 and combination pembrolizumab/lenvatinib in NCT02501096) for consideration outside of the traditional treatment algorithms for EC, and none for serous subtypes specifically (28). Overexpression of DRD2 has been found in gastric cancer, neuroendocrine tumors, glioblastoma, breast cancer, cervical cancer, and colorectal cancer, and have variable association with survival with each histologic subtype (29, 30). Additionally, pharmacological antagonism of DRD2 or knockdown DRD2 expression by siRNA has been shown to have anti-cancer effects, including studies using SEC cells (13, 31–33). Currently, ONC201 is being evaluated in multiple clinical phase I and II trials for several advanced malignancies including leukemias, myelomas, lymphomas, gliomas, and EC, of which preliminary data has revealed a robust pharmacokinetic, pharmacodynamic, and safety profile with evidence of durable tumor regressions and clinical benefit (19, 20). Moreover, in a window of opportunity study, oral ONC201 induced the expression of ATF4 and DR5 in recurrent glioblastoma tissues, suggesting that ONC201 has biological activity in these tumors (34). Importantly, oral ONC201 every 1 to 3 weeks has been overall well tolerated, with no reported grade 3 or 4 drug-associated adverse events (18, 34, 35). Thus, these findings continue to support further development of ONC201 and its more potent analogue ONC206 as therapeutic agents for a variety of cancers, including SEC. Furthermore, exploring the role of the dopamine receptors and its downstream pathways in endometrioid and serous endometrial tumor development as well as identifying new biomarkers related to ONC201 or ONC206 treatment are critical as imipridones to emerge as a promising new class of targeted agents for EC treatment.

## Conclusions

In summary, ONC206 utilizes the ISR in inhibition of cellular proliferation and other vital biologic functions of SEC cells *in vitro*. This preclinical study also expands on our previous work with ONC201 and establishes ONC206 as a more potent alternative in the imipridone family. In the fall of 2019, the Investigational New Drug (IND) for ONC206 was approved by the FDA. Upon determination of its clinical safety profile, ONC206 should be considered in future trials for SECs.

## Data Availability Statement

The raw data supporting the conclusions of this article will be made available by the authors, without undue reservation.

## Author Contributions

YZ, YH, YY, YF, WS, and XZ performed the experiments of cell culture, western blotting, wound healing, and transwell assay. YZ, YH, KT, and AS participated in analyzing and interpreting the data. YZ, SP, and GH wrote the manuscript. VP and JA provided ONC201 and ONC206. CZ and VB-J designed experiments, revised the manuscript, and provided financial support. All authors contributed to the article and approved the submitted version.

## Funding

This work is supported by (1) VLB: American Cancer Society (ACS) Research Scholar Grant - RSG CCE 128826 and (2) VLB: NIH/NCI - R37CA226969.

## Conflict of Interest

VP and JA were employed by the company Oncoceutics.

The remaining authors declare that the research was conducted in the absence of any commercial or financial relationships that could be construed as a potential conflict of interest.
